# Enzymatic corn wet milling: engineering process and cost model

**DOI:** 10.1186/1754-6834-2-2

**Published:** 2009-01-21

**Authors:** Edna C Ramírez, David B Johnston, Andrew J McAloon, Vijay Singh

**Affiliations:** 1 United States Department of Agriculture, Agricultural Research Services, Eastern Regional Research Center, 600 East Mermaid Lane, Wyndmoor, PA 19038, USA; 2 Department of Agricultural Engineering, University of Illinois, 360H, AESB, 1304 W. Pennsylvania Ave, Urbana, IL 61801, USA

## Abstract

**Background:**

Enzymatic corn wet milling (E-milling) is a process derived from conventional wet milling for the recovery and purification of starch and co-products using proteases to eliminate the need for sulfites and decrease the steeping time. In 2006, the total starch production in USA by conventional wet milling equaled 23 billion kilograms, including modified starches and starches used for sweeteners and ethanol production [1]. Process engineering and cost models for an E-milling process have been developed for a processing plant with a capacity of 2.54 million kg of corn per day (100,000 bu/day). These models are based on the previously published models for a traditional wet milling plant with the same capacity. The E-milling process includes grain cleaning, pretreatment, enzymatic treatment, germ separation and recovery, fiber separation and recovery, gluten separation and recovery and starch separation. Information for the development of the conventional models was obtained from a variety of technical sources including commercial wet milling companies, industry experts and equipment suppliers. Additional information for the present models was obtained from our own experience with the development of the E-milling process and trials in the laboratory and at the pilot plant scale. The models were developed using process and cost simulation software (SuperPro Designer^®^) and include processing information such as composition and flow rates of the various process streams, descriptions of the various unit operations and detailed breakdowns of the operating and capital cost of the facility.

**Results:**

Based on the information from the model, we can estimate the cost of production per kilogram of starch using the input prices for corn, enzyme and other wet milling co-products. The work presented here describes the E-milling process and compares the process, the operation and costs with the conventional process.

**Conclusion:**

The E-milling process was found to be cost competitive with the conventional process during periods of high corn feedstock costs since the enzymatic process enhances the yields of the products in a corn wet milling process. This model is available upon request from the authors for educational, research and non-commercial uses.

## Background

The conventional process for wet milling of corn involves chemically pretreating the corn in a solution of sulfurous acid (SO_2 _in water) followed by physical separation of the co-products and starch. This process is very energy and time consuming. Furthermore, it negatively affects the environment due to the high sulfur dioxide requirements during steeping. According to the Environmental Protection Agency, sulfur dioxide is one of the six most common air pollutants in the United States of America [2]. Sulfur dioxide released to the atmosphere is associated with serious respiratory illnesses. At high levels, it particularly affects people with asthma [3]. Also, oxidation of SO_2 _in the presence of other polluting gases in the atmosphere, such as nitrogen dioxide (NO_2_), forms sulfuric acid and causes the formation of acid rain.

Enzymatic wet milling (E-milling) was developed and proposed as an environmentally friendly alternative for conventional corn wet milling [4]. We reported the optimization of conditions for E-milling [5,6] and showed two important advantages to the use of enzymes in a modified two-stage procedure for wet milling; SO_2 _is reduced to levels sufficient to inhibit microbial activity and the time for soaking the corn kernel (steeping) is reduced six fold, from 36 to 6 hours.

The process was developed and tested in the laboratory using a batch process; however, a number of important questions were generated that could not be answered without being tested in a continuous system that included recycling streams. A continuous system with recycle streams cannot be tested on laboratory scale and commercial plant testing is required. Commercial plants are reluctant to evaluate the technology without knowing overall cost benefits of the process. There was a need to investigate the amount and cost of energy savings as a result of reducing steeping time to the much shorter pretreatment time. Furthermore, we needed to know if the cost of the enzyme for the pretreatment was going to make the process uneconomical or if perhaps the savings in energy could balance the cost of the enzyme. Finally, there was the proposed prediction that the recycle of the streams in the continuous process would lower the overall enzyme requirement. All of these questions were answered positively with the help of the process engineering and cost models.

## Methods

### Process model description

The process model was developed using process simulator software (SuperPro Designer^®^) in order to evaluate the continuous production of starch and co-products using E-milling. The model includes processing information such as composition and flow rates of the various process streams, descriptions of the various unit operations, mass and energy balances of each unit operation as well as detailed breakdowns of the operating and capital cost of the facility.

**Figure 1:**
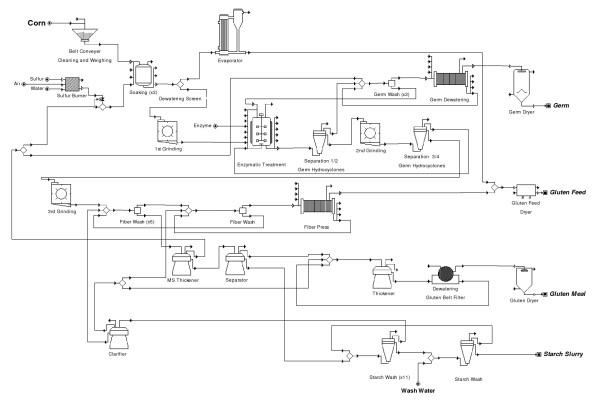
**Simplified flow diagram of the corn E-milling process**. The original model is available upon request from the authors. The model requires the use of SuperPro Designer^®^, Version 7.0, build 17 or later. A free copy of this program can be used to view the model and may be downloaded from the Intelligen website .

Our E-milling model is based on the conventional wet milling model published previously [7] as well as experimental results from the USDA/ARS – Eastern Regional Research Center. The process and model has a capacity of 2.54 million kg of corn per day (100,000 bushels/day) and includes seven main sections: grain handling, pretreatment, enzymatic treatment, germ separation and recovery, fiber separation and recovery, gluten separation and recovery and starch washing and recovery (Figure 1 and Additional file 1). The unit operations in the model are identified by a number ID based on each one of the seven sections (100's for grain handling, 200's for soaking, and so on) and the type of operation (one or two letters to identify equipment). Depending upon the final end product (modified starch, glucose, high fructose corn syrup, ethanol or other fermentation products), downstream differences (after milling) exist in unit operations among wet milling plants. In order for this model to be comparable with most wet milling facilities, it was designed using the universal unit operations found in wet milling plants, up to starch recovery and washing. According to individual user requirements, additional downstream processes could be added if there is a need to model more specific products. Table 1 shows selected unit operations and settings in the process model. Specifics of the process for the conventional wet milling model have been described previously [7]. In the present work, the seven sections of the model are briefly described but only differences between the conventional wet milling and the E-milling process are noted. Table 2 shows the overall material balance for the process. Product yields for the conventional and E-milling models are shown in Table 3. In the E-milling model presented here, we used fairly conservative values for starch yield improvements over the conventional base case model. In the laboratory we have consistently measured more significant increases in starch recovery.

**Table 1:** Overview of selected E-milling process equipment

**Description**	**Detail**
Belt conveyer	55.556 kg/s m loading rate/belt width
Pretreatment tanks	3 tanks 6 h residence time 90% volume 55°C
Sulfur burner	600 ppm of SO_2 _in pretreatment tanks
Enzymatic treatment tank	4 tanks 3 h residence time 75% volume 50°C 4.5 pH
Centrifuge 1	Mill Starch (MS) thickener 7939 l/min throughput 25% (w/w) solids in underflow
Centrifuge 2	Primary separator 3218 l/min throughput 33% (w/w) solids in underflow
Centrifuge 3	Gluten thickener 3112 l/min throughput 17% (w/w) solids in underflow
Centrifuge 4	Clarifier 3945 l/min throughput 28% (w/w) solids in underflow
Hydrocyclone	Last stage of starch washing 4558 l/min throughput 1.3 kg fresh water/kg of dry corn

**Table 2:** Overall material balance for E-milling model

**MATERIALS**	**IN (kg/h)**	**OUT (kg/h)**
Corn (15% water)	106,000	
Air	123,303	123,289
Sulfur	14	
Water	134,624	62,182
Enzyme	12	
Sulfuric acid	49	
Sulfurous Acid		37
Debris		2,539
Dry germ (3% water)		7,211
Gluten feed (10% water)		18,074
Gluten meal (10% water)		6,285
Starch slurry (60% water)		144,385
**TOTALS**	**364,002**	**364,002**

**Table 3:** Corn wet milling product yields (conventional and enzymatic) derived from the process models

**Product**	**Conventional yield (%)^1^**	**Enzymatic yield (%)^1^**
Dry germ	7.7	8.0
Gluten feed (Soak water solids plus fiber)	19.4	18.5
Gluten meal	6.2	6.4
Starch	66.7	67.1

^1^Calculated on a dry weight basis after waste materials (broken corn and foreign matter) are removed.

### Cost model description

The cost model for the E-milling process was developed using the cost analysis capabilities of the software (SuperPro Designer^®^). The model includes economic information such as user-supplied equipment purchase and operating costs of the various unit operations, fixed capital investments for the plant, raw materials and consumable costs as well as detailed breakdowns of the operating and capital cost of the facility. The data for our previous model [7] was obtained from operators of wet milling facilities, equipment suppliers, pricing and cost data reported by trade organizations and government agencies and relevant publications. Inputs from technology suppliers were incorporated into this E-milling study where required. Supplier inputs were obtained from all the major equipment items from suppliers once the process flow diagrams were developed and equipment sizing could be determined. The assembling and analysis of this data was done using the cost estimating program in Superpro Designer^®^, using generally accepted methods for conducting conceptual economic evaluations for industrial processes [8]. Cost levels in both models were adjusted to reflect economic conditions in the first half of 2007.

## Results and discussion

### Process model

#### Grain handling

The corn is received, weighed, cleaned and stored in silos. The silo in our model is sized to hold enough corn for three days of operation.

#### Pretreatment

The steeping step of the conventional process is substituted for a short soaking pretreatment during the E-milling process, long enough to increase the moisture content to 50% in the corn kernel prior to grinding. This pretreatment is very important to preserve the integrity of the germ during grinding. In our model, the corn is soaked in a group of three stainless-steel tanks and held in the soaking solution for a total of 6 h at 55°C. The SO_2 _concentration is 600 ppm for the soaking solution compared with 2000 ppm for the conventional steeping solution. The SO_2 _is used mainly for microbial control in the E-milling process, not as a chemical processing agent. The reduction on sulfur consumption annually is equal to 461,926 kg for a processing plant with a capacity of 2.54 million kg of corn per day. The soaking is done in a semi-continuous countercurrent system, in the same way the steeping is done in conventional wet milling. During the soaking process, about 46% of the soluble solids are removed and carried in the soak water. The soak water is concentrated, mixed with the corn fiber later in the process and dried to produce corn gluten feed. After soaking, the hydrated corn is submitted to a coarse grinding (first degermination) prior to the enzymatic treatment to allow better penetration of the enzyme.

#### Enzymatic treatment

The ground corn along with the overflows of hydrocyclones used for germ separation (except from the A cyclone of primary germ separation unit) is incubated in a reactor tank with a commercial protease (Prosteep™) for 3 h at a controlled temperature of 50°C and pH of 4.5. The amount of enzyme needed for the treatment is based on experimental data and was calculated as 1 mL/kg of corn in the tank (1117–1210 SAPU/kg of corn). The activity of the protease is expressed in Spectophotometric Acid Protease Units (SAPU). One SAPU is the amount of enzyme that liberates one micromole of tyrosine per minute from the casein substrate under the conditions of the assay. Considering the solid content of the 'fresh' corn and the amount of solids being treated (as part of the recycle), the concentration of enzyme in the treatment tank is set to 1.392 g of enzyme/kg of solid material. The process model shows a consumption of 12.12 kg of protease/h or 0.117 g of protease/kg of fresh corn.

During this step, the protease hydrolyzes the protein matrix (gluten) that surrounds the starch granules. The enzymatic treatment disrupts starch-gluten interactions so that the starch and gluten can be separated. The proteolysis must not be so extensive as to completely degrade the gluten matrix (preventing gluten recovery), which is why not all proteases found to allow starch recovery are acceptable for this process. The proteolysis treatment and the conventional process using sulfites both allow the starch to be isolated by disrupting the starch-gluten interactions; however, the specific chemical mechanism of the two pretreatments is not identical and there can be some additive benefits of using them together [6].

#### Germ separation and washing

The enzyme-treated corn continues in the process in order to separate the oil-rich corn germ from the starchy slurry using four sets of hydrocyclones. The separation is based on the lower density of the germ, compared with the density of the slurry. The overflows of all hydrocyclones, with the exception of the first set, are recycled to the enzymatic treatment tank to optimize the purity of the germ recovered. The pure germ is washed, dewatered and dried as in the conventional process. The underflow of the last set of hydrocyclones (separation 4) continues the co-product separation process. The dry germ is produced in our model at a rate of 7211 kg/h and it contains a higher protein but lower lipid content than the conventional process (Table 4). The difference in composition is due to the increase of soluble solids in the stream with the conventional process.

**Table 4:** Protein content, lipid content and unit price of the co-products derived from the wet-milling process models (conventional and enzymatic)

**Products**	**Conventional**			**Enzymatic**		
	**% Protein**	**% Lipid**	**($/kg)**	**% Protein**	**% Lipid**	**($/kg)**
Dry germ	11.6	45.0	0.296	12.6	44.0	0.295
Gluten feed^1^	16.2	2.1	0.080	16.1	2.1	0.080
Gluten meal	66.3	2.0	0.400	65.5	2.0	0.396

Compositions are on a dry weight basis.

^1^Soak water solids plus fiber

#### Fiber separation and recovery

This stage of the process remains as the conventional treatment where the degermed corn slurry is passed over the grit screen to separate water, loose starch and gluten (together known as mill starch) from the fiber and bound starch and gluten. The mill starch is sent further in the process, for the separation of gluten and starch. The remaining solids are finely ground to complete the dispersion of the starch and the ground slurry is washed and separated in countercurrent fashion over a set of screens. The clean fiber is dewatered by a screen and a screw press to a final moisture of 60%. This fiber is combined with the concentrated soak water, dried to 10% moisture and sold as corn gluten feed. The corn gluten feed flow is 18,074 kg/h and has approximately the same protein content compared with traditional wet milling (Table 4).

#### Gluten separation and recovery

As in the conventional process, the gluten is separated from the starch by density differences in a series of three centrifuges where the underflow of the middle one, known as the primary separator, is sent to the starch washing process. The last centrifuge (gluten thickener), along with a rotary vacuum belt filter and a ring dryer, concentrates the gluten to a final moisture of 10%. The gluten is sold as corn gluten meal. The final corn gluten meal (6,285 kg/h in our model) has approximately the same protein content on a dry weight basis as the conventional process (Table 4).

#### Starch washing and recovery

The washing and recovery of the starch is done in 12 stages in a countercurrent fashion, as it is done in the conventional wet-milling process. This is the only part of the process where fresh water is used to wash the product. The water usage is essentially the same for E-milling and conventional processes (2.3 kg water/kg starch produced). The final starch slurry (144,385 kg/h) contains 60% moisture content with less than 1% of impurities.

### Cost model

#### Equipment and capital costs

The estimated capital cost for the construction of a wet milling plant area processing 100,000 bushels of corn per day is estimated to be approximately $79,300,000 while the capital cost of constructing an E-milling processing area of the same capacity is estimated to be $74,900,000. These differences in capital costs are attributable to the following reasons; the use of enzymes reduces the amount of sulfur required for steeping as well as the length of soaking time. These differences result in a capital cost reduction in the soaking area of approximately $9,670,000 which is partially offset by the requirements for the pretreatment tanks now required at a cost of $3,670,000. Table 5 shows the capital costs by section of the process.

**Table 5:** Capital costs by section

**Section**	**Conventional^1^**		**Enzymatic**	
	**%**	**(US$ × 100)**	**%**	**(US$ × 100)**
Grain handling	8.2	6,500	8.7	6,500
Steeping (or pretreatment)	22.3	17,700	10.7	8,000
Enzymatic treatment	0	0	4.9	3,700
Germ separation	13.5	10,700	14.4	10,800
Fiber separation	23.7	18,800	25.9	19,400
Gluten separation	27.1	21,500	30.0	22,500
Starch washing	5.2	4,100	5.5	4,100
**TOTAL**	**100.0**	**79,300**	**100.0**	**75,000**

^1 ^From Ramirez et al 2008 [7].

Furthermore, the use of enzymes in the process has the effect of keeping more of the soluble solids with the gluten, fiber and germ and less with the corn soak liquor. The higher soluble loading translates into more material being processed in the gluten, fiber and germ areas and consequently larger equipment capacities and costs. Overall, the combination of the above factors results in a lower capital cost for the E-milling facility of about $4,400,000 or about a 5.5% reduction in costs over a conventional wet milling line.

#### Operating costs

The operating costs for an E-milling facility are similar to the operating costs for a conventional wet milling facility. We have estimated that the cost of producing a clean starch slurry for further processing by both the wet milling process and the E-milling process would be about $0.193 per kilogram.

The reductions in the capital cost of an E-milling facility are described above. When these savings are spread over a 10-year period the operating cost is reduced by approximately $440,000 per year. Reductions in insurances, taxes and maintenance fees, which are all related to the estimated capital cost savings, provide a reduction in costs of approximately $190,000 per year.

The throughput to the evaporator is limited to a concentration of 50% solids in the syrup leaving the evaporator. Since the concentration of solids in the steep water to the evaporator is lower in the E-milling case, a higher volume of water can be removed in the evaporator at a lower cost than would be achieved in a dryer which results in an additional cost saving of about $40,000 per year.

The lower concentration of sulfur needed results in cost savings of $15,000 per year while the inclusion of the enzymes required for the process adds $1,440,000 per year to the operating costs. The need for sulfuric acid in the enzymatic process for pH adjustment adds $49,000 per year to the operation costs.

#### Product values

The starch co-products produced in a wet milling facility include two protein-based animal feeds (corn gluten meal and corn gluten feed) that are valued for their protein content and a third co-product, corn germ, whose price is a function of its protein content and its lipid content [9]. Table 4 shows the protein and lipid content on a dry weight basis in the co-products for both processes. In E-milling, the protein content slightly decreases for corn gluten meal and corn gluten feed and increases for corn germ. The lipid content in the germ decreases by 1%. The difference in composition of the co-products creates a difference in the unit price for co-products. The unit prices were calculated using the method describe by Johnston et al [9].

The relative quantities of the co-products also vary from a conventional wet milling facility to an E-milling facility. The result is that the net value of the co-products is 0.3% greater for an E-milling facility than a conventional wet milling plant.

#### Annual and unit production costs

Corn starch, as a water slurry, is the principal product of the wet milling and the E-milling processes. A comparison of the economics of these processes is then achieved by comparing the unit cost of producing the starch in each case.

Unit production costs are calculated by prorating the total annual starch production costs over the annual production volume. Annual production costs for the production of starch slurry are calculated by adding together all the annual operating costs to produce the starch slurry and its co-products and then reducing this number by the income received from the value of the co-products of the starch production. The annual production costs include a depreciation allowance of 10% of the capital cost which is based on a 10-year effective operating life for the facility with no salvage value at the end of its life, and the operating costs described in Table 6.

**Table 6:** Annual operating and production costs

	**Conventional**		**Enzymatic**	
	Material flow	Annual cost	Material flow	Annual cost
	Metric ton/year	(US$x1000)/year	Metric ton/year	(US$x1000)/year

**Operating costs**				
Raw materials				
Corn – kg	839,520	111,018	839,520	111,018
Enzyme				1,440
Other raw materials		396		418
Depreciation		7,933		7,494
Facility related costs		3,467		3,275
Utilities		12,550		12,508
Operations labor		1,980		1,980

Total operating costs		137,344		138,134

**Co-product credits**				
Corn gluten meal	48,090	19,255	49,788	19,696
Corn gluten feed	150,439	12,071	143,216	11,414
Corn germ	55,684	16,482	57,117	16,855

Total co-product credits		47,808		47,965

**Annual starch production^1^**	463,150	89,536	466,632	90,169
Unit starch production cost ($/kg)		$0.19332		$0.19323

^1 ^Dry basis

#### Cost comparison with conventional wet milling

The E-milling process can be economically competitive with the conventional wet milling process and our models have indicated a slight, but not significant, cost advantage to the E-milling process over the conventional wet milling process under the economic conditions that existed in the first half of 2007.

**Figure 2:**
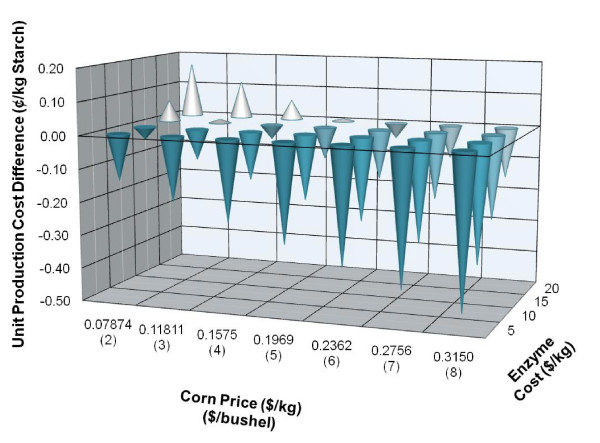
**Impact of corn and enzyme price on starch unit production cost**. The data reflects the difference between the E-milling minus the conventional process. A negative difference indicates a reduction in unit production cost.

Using the model, historic values and future estimates for corn prices ($0.09 to $0.32/kg or $2 to $8/bushel) and the estimated range of enzyme cost ($5 to $20/kg), the unit production cost of starch for the E-milling and the conventional processes was calculated and the differences compared. The differences (E-milling unit production cost minus conventional unit production cost) are summarized in Figure 2 and clearly show that under certain corn and enzyme prices the E-milling process can be either more or less economical relative to the conventional process. Under the current enzyme price ($15/kg) and corn cost ($0.132/kg or $3.36/bushel), there is only a slight economic advantage of the E-milling process; however, as the cost of corn increases and/or the price of enzyme decreases, the economic advantage of the E-milling process quickly becomes significant over the current conventional process.

## Conclusion

A Technical Cost Model was developed for an enzymatic corn wet milling processing plant with a capacity to process 2.54 million kg of corn per day. This model was used as a tool to understand the differences between the E-milling and conventional wet milling processes, and the cost issues associated with it. We used the model to conduct sensitivity studies using modifications in the price of corn and enzyme. The model allows the user to predict the impact of those modifications in the operating, annual and unit production costs. Our comparison shows that due to the significant recycle of enzyme within the process (quantified using the process model), a significant reduction in the quantity of enzyme necessary over a batch process is possible even if some unaccounted activity losses due to adsorption or inactivation were to occur. It was also found that under current corn and enzyme costs, the E-milling process is slightly more economic on a unit starch production cost; however, it was also shown that under high corn and/or reduced enzyme costs the process can be significantly more economical than the conventional process.

Additionally, the reduction in sulfur consumption was found to be 461,926 kg/year for the model size of 2.54 million kg/year (100,000 bu/day). If adapted industry wide within the United States of America, this would translate into a reduction of 12.6 million kg of sulfur per year.

This model is available upon request from the authors for educational uses and non-commercial research to study the enzymatic wet milling process and to show the impact of changes in the costs of starch, enzyme and co-products. It is not intended to replace a customized process design package. The model requires the use of SuperPro Designer^®^, Version 7.0, build 17 or later. A free copy of this program can be used to view the model and may be downloaded form the Intelligen website .

## Competing interests

The authors declare that they have no competing interests.

## Authors' contributions

ECR developed the E-milling processing model, evaluated the results and drafted the manuscript. DBJ and VS developed the E-milling process and conducted laboratory, pilot plant and commercial plant trials to obtain the process data necessary for the model. AJM obtained the material and equipment cost data and conducted the economical analysis of the model. All authors suggested modifications to the draft, commented on several preliminary versions of the text and approved the final manuscript.

## Supplementary Material

Additional file 1 **Enzymatic milling.** Detailed flow diagram of the SuperPro Designer^® ^process model. Click here for file

## References

[B1] Corn Refiners Association Inc (2007). Corn Annual Washington DC.

[B2] United States Environmental Protection Agency (2000). SO2 How Sulfur Dioxide Affects the Way We Live and Breathe.

[B3] Boushey H (1982). Asthma, sulfur dioxide and the Clean Air Act. West J Med.

[B4] Johnston DB, Singh V (2001). Use of proteases to reduce steep time and so_2 _requirements in a corn wet-milling process. Cereal Chem.

[B5] Johnston DB, Singh V (2004). Enzymatic milling of corn: optimization of soaking, grinding, and enzyme incubation steps. Cereal Chem.

[B6] Johnston DB, Singh V (2005). Enzymatic milling product yield comparison with reduced levels of bromelain and varying levels of sulfur dioxide. Cereal Chem.

[B7] Ramírez EC, Johnston DB, McAloon AJ, Yee W, Singh V (2008). Engineering process and cost model for a conventional wet milling facility. Ind Crops Prod.

[B8] Association for the Advancement of Cost Engineering International (1990). Conducting Technical and Economic Evaluations in the Process and Utility Industries. AACE Recommended Practices and Standards.

[B9] Johnston DB, McAloon AJ, Moreau RA, Hicks KB, Singh V (2005). Composition and economic comparison of germ fractions from modified corn processing technologies. JAOCS.

