# Supplementary material for: Enzymatic corn wet milling: engineering process and cost model
Source: Biotechnol Biofuels. 2009 Jan 21;2:2. doi: 10.1186/1754-6834-2-2 (PMC2633287; doi:10.1186/1754-6834-2-2)
Supplement: Additional file 1 — Enzymatic milling. Detailed flow diagram of the SuperPro Designer® process model. [file 1754-6834-2-2-S1.pdf]

Enzymatic Milling

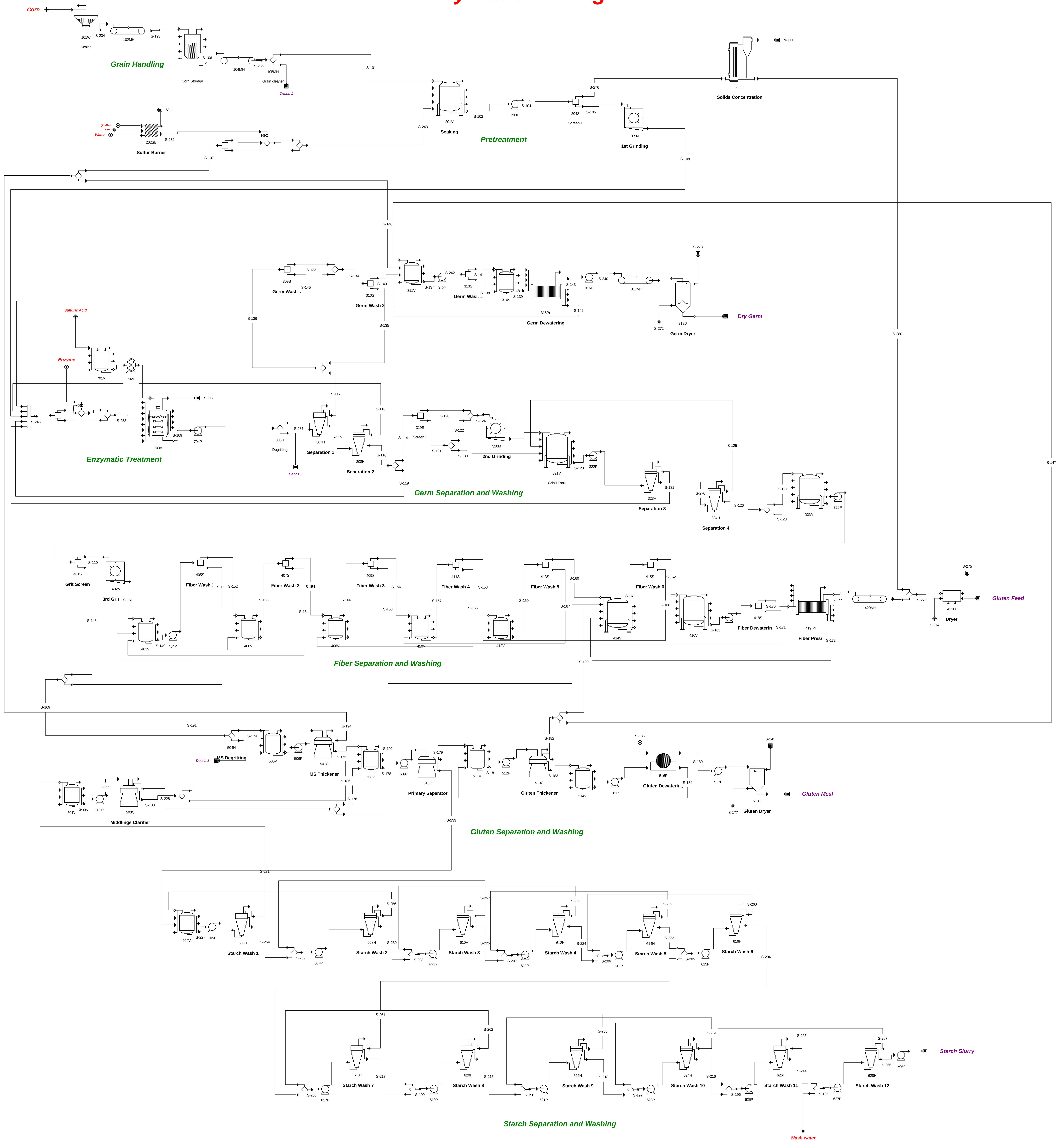

DISCLAIMER

This simulation was developed for non-commercial research and educational uses only. The authors and the Agricultural Research Service of the US Department of Agriculture do no accept responsibility for its accuracy or for conclusions or decisions derived from it.
For specific applications of this simulation, users should contact the authors for more detailed information, limitations and scope of the model.

U. S. DEPARTMENT OF AGRICULTURE
AGRICULTURAL RESEARCH SERVICE
EASTERN REGIONAL RESEARCH CENTER
WYNDMOOR, PA 19038

100,000 Bushels/Day Corn Enzymatic Model

CROP CONVERSION SCIENCE AND ENGINEERING UNIT

LEAD SCIENTIST	David Johnston
PROCESS ANALYSIS	Edna Ramirez
ECONOMIC ANALYSIS	Andrew McAloon
LATEST REVISION	December 15, 2008
